# The Effect of Dissemination Pathways on Uptake and Relative Costs for a Transdiagnostic, Self-guided Internet Intervention for Reducing Depression, Anxiety, and Suicidal Ideation: Comparative Implementation Study

**DOI:** 10.2196/34769

**Published:** 2022-05-06

**Authors:** Philip J Batterham, Amelia Gulliver, Ella Kurz, Louise M Farrer, Christiaan Vis, Josien Schuurmans, Alison L Calear

**Affiliations:** 1 Centre for Mental Health Research The Australian National University Canberra Australia; 2 Faculty of Behavioural and Movement Sciences Vrije Universiteit Amsterdam Amsterdam Netherlands; 3 GGZ InGeest Amsterdam Netherlands

**Keywords:** implementation, mental health, adherence, uptake, internet

## Abstract

**Background:**

Self-guided web-based programs are effective; however, inadequate implementation of these programs limits their potential to provide effective and low-cost treatment for common mental health problems at scale. There is a lack of research examining optimal methods for the dissemination of web-based programs in the community.

**Objective:**

This study aimed to compare the uptake, reach, relative costs, and adherence associated with 3 community-based pathways for delivering a low-intensity web-based transdiagnostic mental health program. The 3 dissemination pathways were social media advertising, advertising in general practice, and advertising in pharmacies.

**Methods:**

Participants were recruited on the web, from general practices, or from community pharmacies; completed a screener for psychological distress; and were offered the 4-week *FitMindKit* program—a 12-module psychotherapeutic intervention. Uptake was defined as the number of participants who enrolled in the web-based program; reach was defined as the rate of uptake per exposure; and costs were calculated based on staff time, equipment, and advertising. Adherence was assessed as the number of modules of *FitMindKit* completed by the participants.

**Results:**

Uptake comprised 1014 participants who were recruited through the 3 dissemination pathways: on the web (991/1014, 97.73%), in general practice (16/1014, 1.58%), and in pharmacy (7/1014, 0.69%). Reach was highest for social media: 1 in every 50 people exposed to web-based advertising took up the intervention compared with 1 in every 441 in general practitioner clinics and 1 in every 1708 in pharmacies. The dissemination cost was US $4.87 per user on social media, US $557 per user for general practitioner clinics, and US $1272 per user for pharmacy dissemination. No significant differences in adherence were observed between the conditions, whereas all pathways showed an underrepresentation of men and linguistic diversity.

**Conclusions:**

The web-based dissemination pathway was the most efficient and cost-effective for delivering a self-guided internet-based mental health program to people in the community. More research is needed to identify how best to engage men and those with culturally diverse backgrounds in web-based interventions.

**Trial Registration:**

Australian New Zealand Clinical Trials Registry ACTRN12618001688279; https://www.anzctr.org.au/Trial/Registration/TrialReview.aspx?id=376113

## Introduction

### Background

Depression and anxiety are common mental health problems, with 10% to 15% of the global population experiencing a lifetime mood disorder and 10% to 15% experiencing a lifetime anxiety disorder, and depression and anxiety prevalence worldwide were 3152 and 4802 per 100,000, respectively, in 2020 [1-4]. They can cause significant disability burden and affect many domains of life, including physical, social, work, and education [5-7]. Despite this considerable impact, more than half of people experiencing mental disorders worldwide do not seek professional help at onset, typically delaying care for many years, with <10% receiving adequate treatment [8-10]. A number of factors have been identified as contributing to the low levels of help seeking in the community, including the accessibility of services (location and cost), mental health stigma, and mental health literacy [11-13]. Web-based self-guided mental health interventions have been proposed as a way of addressing low help-seeking rates, particularly as they can circumvent some of the barriers by providing private, low-cost access to treatment [14]. Consequently, community-based provision of evidence-based, low-intensity, self-guided web-based mental health programs may assist in increasing treatment access for people who would not otherwise seek help [15,16].

In addition to being prevalent, depression and anxiety also have high rates of comorbidity between them and with other mental disorders [17]. Transdiagnostic therapeutic approaches involve treating multiple conditions simultaneously by targeting the core mechanisms underlying comorbid conditions (eg, negative thinking patterns) rather than diagnosis-specific treatment [18]. Transdiagnostic programs are highly suited for web-based dissemination and have been demonstrated to be effective in reducing symptoms of depression and anxiety [19,20]. In addition, self-guided interventions that do not require clinician support have been found to be safe and effective in reducing the symptoms of depression and anxiety [15,21,22], particularly for people with mild to moderate symptom severity. Self-guided interventions require minimal resources and are consequently highly scalable [23], although clinician-guided programs typically show stronger effects [15] and greater acceptability [24]. Despite the availability and accessibility of effective self-guided interventions, community uptake of web-based mental health programs remains poor [14,25]. For example, a naturalistic study examining uptake in the community of a web-based depression program found that half of all visitors to the program’s website (N=194,840) did not register for the program [26]. Therefore, ensuring that effective programs are implemented in the community is critical to realizing the full potential of web-based mental health programs.

The field of implementation science focuses on how best to implement evidence-based treatments into health care services [27]. In addition to low uptake within the community, the implementation of web-based mental health programs in clinical service settings remains a challenge [16,28]. Although there is strong evidence that both self-guided and clinician-guided programs are effective for preventing and treating depression and anxiety [16,28-31], only 14% of new evidence-based interventions enter routine practice [32,33]. In addition, successful programs are typically only implemented several years after establishing their clinical efficacy [32,34].

The uptake of web-based mental health programs within primary care (eg, via general practitioners [GPs]) is emerging but has been relatively slow [35-37]. Previous research conducted in Australia [38] examined the potential of recruiting people with type 2 diabetes and depression into a web-based mental health program. This study found that recruitment through general practice (n=24 participants) was unsuccessful compared with going through registries and word of mouth (n=196) or web-based recruitment methods (eg, Facebook; n=520) [38]. The authors identified staff attitudes toward internet interventions as a key barrier to recruitment through primary care in Australia [38]. Other potential community-based settings that offer promise in increasing uptake of internet interventions include community pharmacies (ie, pharmacies outside of hospitals and other care facilities), which have previously been investigated as a potential avenue for recruitment of participants into community health care trials [39]. However, little is known about the potential of pharmacies to act as a pathway to treatment for web-based mental health services. Dissemination of internet interventions directly to end users through web-based methods, such as social media marketing or digital apothecaries (directories of evidence-based programs), is more common and may be better suited to the modality and self-directed nature of these interventions [40].

The gap between the evidence and uptake of web-based mental health programs is exacerbated by a paucity of translational research into how best to implement these programs within traditional health care settings and directly in the community. Conducted as part of a broader project targeting implementation [41], this project addresses this research gap by testing different pathways for disseminating the *FitMindKit* e-mental health program to people experiencing symptoms of depression and anxiety in the community. *FitMindKit* is a self-guided, web-based mental health program that delivers low-intensity transdiagnostic cognitive behavioral therapy over 4 weeks via 12 modules comprising brief videos and self-directed exercises [42,43]. The program has been found to be effective in a community-based trial for improving symptoms of depression, panic disorder, and social anxiety [44]. In this study, dissemination was tested as a specific implementation strategy [45] to increase intervention reach and engage more people in need of treatment with a low-threshold web-based intervention. We compared the 3 dissemination pathways to identify which had the greatest reach, uptake, and adherence and lowest costs.

### Hypotheses

We hypothesized that direct social media advertising on the web would be associated with greater uptake (number of participants screened and enrolled in the web-based program) than recruitment via advertising using posters or tablet computers in general practices and pharmacies. It was anticipated that the relative reach (ratio of uptake to exposure) would be highest in the social media pathway, whereas the cost per enrolled participant would consequently be lower in this pathway. In addition, we hypothesized that engagement with the program (based on adherence; module completion) would be lowest among people recruited through social media, as this group had the least possibility of human contact in the recruitment process [46]; that is, the higher uptake on the web may come at the expense of engagement with the intervention. We did not have any specific hypotheses regarding outcomes for the pharmacy pathway, as this pathway has not been investigated previously in e-mental health implementation research. We also examined the diversity of participants recruited via each dissemination arm to assess the relative inclusivity of each dissemination pathway as an exploratory analysis with no specific hypotheses.

## Methods

### Study Design and Participants

This project was part of a larger implementation trial [41] and was designed to test the effectiveness of three different dissemination pathways—(1) social media, (2) general practices, and (3) pharmacies—in delivering an e-mental health program (*FitMindKit*) to people in the community with elevated symptoms of psychological distress. Dissemination methods were tailored for each pathway, with different approaches to inviting participants to complete a brief screening measure assessing psychological distress. The tailoring involved consulting with clinic and pharmacy staff about the most appropriate ways of engaging their clients and optimizing social media advertising based on past evidence [47].

Adults aged ≥18 years who were screened with elevated psychological distress based on scores of 8 to 17 on the Distress Questionnaire 5 (DQ5) [48], which primarily reflects symptoms of mood or anxiety disorders [49], were eligible and invited to use *FitMindKit* for a period of 4 weeks. The DQ5 is a brief screening measure comprising 5 items describing symptoms of common mental disorders. Participants endorsed the frequency of each item over the past 30 days on a 5-point scale ranging from never (1) to always (5). Total scale scores ranged from 5 to 25; low scores (5-7) indicate no or low psychological distress, scores of 8 to 17 indicate a moderate level of psychological distress, and scores of 18 to 25 indicate a high level of psychological distress, based on quartiles from a previous validation study [48]. Those who were ineligible because they were in the low-risk category (DQ5 score=5-7) were provided with feedback to continue monitoring their psychological well-being and with help resources to access if their symptoms changed. Those who were ineligible because they were in the high-risk category (DQ5 score=18-25) were provided with feedback strongly encouraging them to seek help from a health professional and contact details for face-to-face, telephone-based, and web-based mental health resources and services.

Participants completed brief pre- and postprogram questionnaires and received weekly email reminders to use *FitMindKit*. The study was conducted from July 2018 to December 2020; however, the social media arm was only active from October 2018 to August 2019; therefore, only data from this period were used for comparisons for all pathways.

### Ethics Approval

The ethical aspects of this research were approved by the Australian National University Human Research Ethics Committee (protocol number 2017/911).

### Recruitment

#### General Practice and Pharmacy

General practices and pharmacies located in the Australian Capital Territory were invited to participate in the study via an invitation letter, followed up by a telephone call and an in-person visit. Invitations were sent to 49 general practices and 41 pharmacies. Of these, 10% (5/49) of general practices and 12% (5/41) of pharmacies participated in the study, with the remainder either not responding after 2 follow-ups or declining because of lack of interest, lack of time, or limited infrastructure (eg, no Wi-Fi connection). Promotional posters and flyers were provided to participating general practices and pharmacies to promote the study to patients and customers. A tablet computer was also provided in the waiting areas of general practices and pharmacies, where interested participants could read information about the study, provide their consent to participate, and complete a screening survey to assess their eligibility for the study.

During the project, the researchers actively engaged with staff in the participating general practices and pharmacies to identify and remediate any barriers or difficulties they faced in implementing the project. Key staff in practices and pharmacies were also asked to complete a brief measure at the beginning and end of the project to assess their experience with the implementation process.

#### Social Media

Participants were recruited to the social media pathway of the study via Facebook and Instagram advertisements. To ensure that the geographical catchment area for this pathway was consistent with that of the GP clinics and pharmacies, the advertisement specifically targeted adults living in the Australian Capital Territory aged ≥18 years. It read, “Want to learn more about your mental health and well-being? Complete a brief survey and 4 week online program. Participants needed for a mental health study.” A series of images that were used on a rotational basis accompanied the advertisements and featured natural scenes (eg, trees, plants, and clouds). Participants who clicked on the advertisement received a full information sheet with details of the program, were screened, completed baseline measures, and then randomly allocated to receive *FitMindKit* (991/1986, 49.9%) or an attention control website—*HealthWatch* (995/1986, 50.1%)—for 4 weeks. This study only included data from participants who were allocated to the *FitMindKit* condition and excluded participants who were allocated to the control condition.

### Procedure

#### Overview

**Figure 1:**
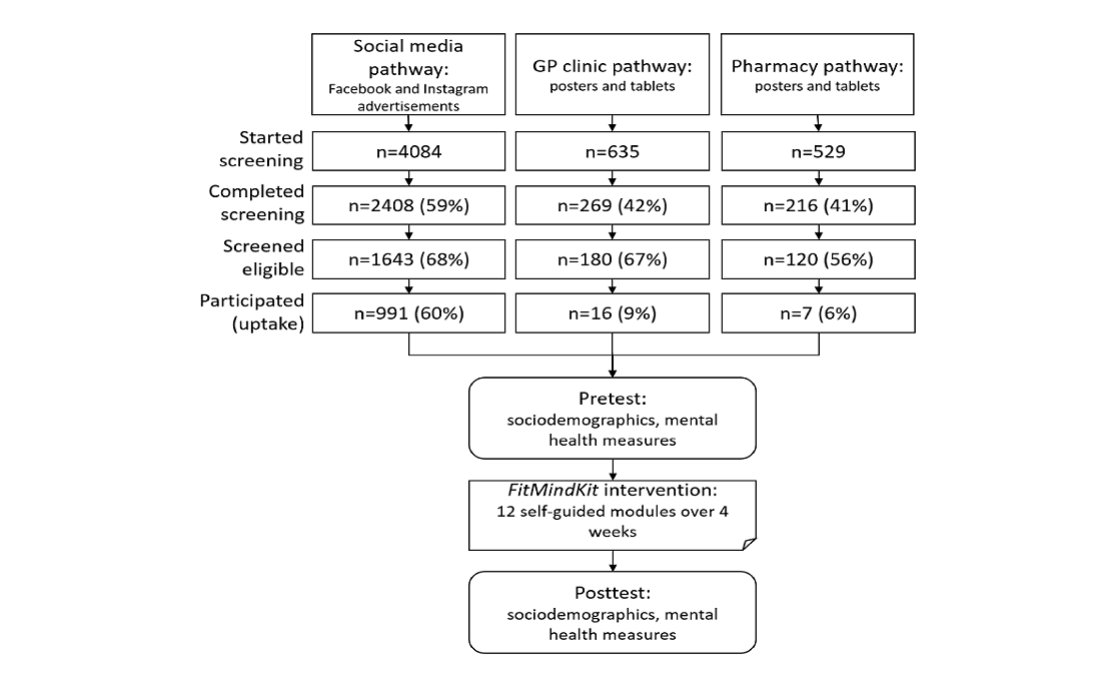
Flow of participants in the study across the 3 dissemination pathways. GP: general practitioner.

The flow of participants through the study is summarized in Figure 1. Data for the current analyses were obtained from screening and pretest assessments only. After obtaining informed consent and screening, participants were directed to create an account in the project portal using an email address and password. Within the portal, all participants completed a baseline questionnaire, irrespective of the dissemination pathway. Those in the GP and pharmacy pathways were offered the intervention, and those in the social media pathway were randomized to receive either the intervention or an attention control condition, although only data from participants who received the active intervention were included in the current analyses. Participants were sent a weekly reminder email to encourage them to engage with *FitMindKit*. Following the 4-week period, participants were sent an email inviting them to complete the postintervention assessment. They received 2 reminder emails if they did not complete the postintervention assessment after 1 and 2 weeks.

#### FitMindKit Program

*FitMindKit* is a transdiagnostic web-based program comprising brief (10-minute) therapeutic modules to reduce the symptoms of common mental disorders. Each module comprises a brief video (2-6 minutes) using a series of professionally designed and animated fictional characters, some of whom share their experience of a mental health problem and some of whom describe specific therapeutic techniques. Each module includes a written transcript and a subsequent written exercise that encourages the person to practice the described therapeutic technique. The program was adapted for this trial from a previous version of the intervention that was tailored to symptom profiles, with the tailoring removed because of a lack of increased efficacy [42]. The 12 modules of the current version of the program provide techniques predominantly based on cognitive behavioral therapy. The program included 8 core transdiagnostic modules (psychoeducation, getting help and support, cognitive reframing, problem-solving, mindfulness, managing relationships, exercise and diet, and sleep hygiene). It also contained 4 modules designed for specific mental health problems but focused on therapeutic targets broadly relevant to internalizing psychopathology, including 2 depression modules (behavioral activation and reducing rumination), 1 anxiety module (exposure), and 1 suicidality module (distress tolerance). The ordering of the program was self-directed, except that the participants were required to complete the psychoeducation module first. Participants had access to all 12 *FitMindKit* modules for 4 weeks.

#### Measures

*Uptake* was assessed as the number of participants who enrolled in the intervention and whether they completed any of the modules. *Reach* was assessed as the ratio of people who took up the intervention to those exposed to information or advertisements for the study within each pathway, based on estimates provided by the social media platform (Facebook and Instagram) and estimates of patient or customer throughput provided by the clinics and pharmacies. *Relative uptake* was examined by assessing the number of people taking up the intervention within each pathway as a proportion of the reach of that pathway. Another indicator of reach, *inclusion*, was assessed on the basis of diversity in the characteristics of participants from each of the dissemination pathways; that is, no demographic groups were omitted from any of the pathways, and user characteristics in each pathway were largely consistent with each other and with the general population. Demographic characteristics included gender (male, female, or other gender), age (18-25, 26-35, 36-45, 46-55, 56-65, or >66 years), level of education (primary school, some secondary school or year 10 equivalents, year 12, Certificate Level I-IV, diploma or associate degree, bachelor’s degree, graduate diploma or graduate certificate, master’s degree, and doctoral degree), employment status (full-time, part-time or casual, unemployed, or not working because of study, maternity leave, or retirement), and language spoken at home (English only, English and another language, or another language only). Clinical characteristics included symptoms of depression (Patient Health Questionnaire-9 score, ranging from 0 to 27) [50], generalized anxiety (Generalized Anxiety Disorder-7 score, ranging from 0 to 21) [51], social anxiety (social anxiety disorder screener, range 0-16) [52], panic disorder (Panic Disorder Screener, range 0-16) [52], and suicidal thinking (Suicidal Ideation Attributes Scale, range 0-50) [53].

*Costs of implementation* were calculated on the basis of the costs of research staff time to engage each dissemination pathway and the costs of marketing (social media advertisements, posters, flyers, and tablets) associated with each pathway. The costs are provided in Australian dollars. *Adherence* was assessed as the number of modules of *FitMindKit* completed by the participants.

#### Data Analysis

Descriptive analyses were used to compare uptake, reach, and costs. Adherence (module completion) was compared between pathways using independent-sample 2-tailed *t* tests. The diversity of participants was assessed by comparing participant characteristics between the 3 pathways using chi-square statistics for categorical variables and the Welch test for continuous variables to account for homogeneity of variance between pathways. SPSS (version 26; IBM Corp) was used for statistical analyses.

## Results

### Uptake, Reach, and Inclusion

A total of 1014 participants were recruited into the *FitMindKit* program through three dissemination pathways: social media (991/1014, 97.73%), general practice (16/1014, 1.58%), and pharmacy (7/1014, 0.69%). A total of 1986 participants were recruited in the social media setting, and 991 (49.9%) were allocated to the control condition. Figure 1 shows that the rate of screening completion was highest in the social media pathway. Eligibility rates were similar across pathways; however, stark differences were seen in the rates of participants starting the intervention, with social media participants being considerably more likely to start the intervention.

The total potential exposure for the 3 pathways was assessed on the basis of the reach of social media advertising and estimates provided by the clinics and pharmacies of annual patient or customer numbers. Staff at the GP clinics estimated a total of 47,418 annual visits. On the basis of the Australian average of 5.6 annual visits per person [54], this represents 8468 individuals. Pharmacy visits were harder to estimate but based on the adult population of the catchment area (n=306,000) and the number of community pharmacies in the region (n=80), it was estimated that 14,345 adults visited 1 of the 5 pharmacies annually under the assumption that 75% of the adult population visited a community pharmacy. Reach of the social media advertisement was 98,135 individuals, with 448,797 impressions of the advertisements delivered.

On the basis of these estimates, 1 in every 50 people exposed to the social media advertising took up the intervention (991/49,068, 2.02% of individuals accounting for the control condition), compared with 1 in every 441 in GP clinics (16/7057, 0.22% of patients over 10 months) and 1 in every 1708 in pharmacies (7/11,953, 0.06% of customers over 10 months). Given the low rates of uptake in GP or pharmacy settings, we also examined the rates of screening completion as a proportion of exposed individuals as an alternative metric of reach. Screening completion rates were 4.91% (2408/49,068) in social media, 3.81% (269/7057) in GP clinics, and 1.81% (216/11,953) in pharmacies.

Table 1 presents the characteristics of the participants in each of the 3 dissemination pathways. There were no demographic groups that were poorly represented in any of the pathways, except there were few participants who spoke a language other than English, and there was an underrepresentation of men. When comparing the inclusion of demographic subgroups across the 3 pathways, there was some evidence that the social media sample trended toward being younger, more women, and more likely to speak only English. However, the only significant demographic difference was in employment status (*χ^2^_2_*=12.3; *P*=.002), indicating that participants in the social media pathway were more likely to be employed in work than those in the other pathways. For the GP and pharmacy pathways, all of those not working were classified as *not in the labor force* (eg, retired, student, and maternity leave) rather than unemployed, whereas in the social media pathway, 31.8% (69/217) of those not working were unemployed. In terms of mental health symptoms, there were significant differences in generalized anxiety symptoms, with GP participants having less severe anxiety than the other pathways (Welch test=7.00; *P*=.01), with a similar trend observed in panic symptoms. There were no significant differences in depression symptoms or suicidal ideation.

**Table 1:** Characteristics of participants in the 3 pathways (N=1014).

Characteristics		Social media (n=991)	GP^a^ clinic (n=16)	Pharmacy (n=7)	Chi-square (*df*)		Welch test		*P* value	
Gender (female), n (%)		841 (84.9)	11 (68.8)	6 (85.7)	3.1 (1)		N/A^b^		.21	
**Age group (years), n (%)**						8.9 (2)		N/A		.06
	18-35	413 (41.7)	4 (25)	2 (28.6)						
	36-55	418 (42.2)	6 (37.5)	2 (28.6)						
	≥56	160 (16.1)	6 (37.5)	3 (42.9)						
**Educational attainment, n (%)**						2.7 (1)		N/A		.26
	Less than bachelor’s degree	437 (44.1)	6 (37.5)	1 (14.3)						
	Bachelor’s or higher	558 (56.3)	10 (62.5)	6 (85.7)						
**Employment, n (%)**						12.3^c^ (1)		N/A		.002^c^
	Full-time or part-time	774 (78.1)	7 (43.8)	4 (57.1)						
	Unemployed or not in labor force	217 (21.9)	9 (56.3)	3 (42.9)						
**Language spoken at home, n (%)**						5.01 (1)		N/A		.08
	English only	902 (91)	12 (75)	6 (85.7)						
	Other languages	89 (9)	4 (25)	1 (14.3)						
PHQ-9^d^ depression score, mean (SD)		10.60 (4.95)	8.94 (5.77)	9.57 (3.46)	N/A		0.90		.36	
GAD-7^e^ anxiety score, mean (SD)		8.16 (4.38)	4.75 (3.54)	9.14 (4.56)	N/A		7.00^c^		.01^c^	
PADIS^f^ panic score, mean (SD)		3.20 (2.97)	1.63 (2.36)	4.14 (3.34)	N/A		3.57		.06	
SOPHS^g^ social anxiety score, mean (SD)		6.17 (3.80)	4.88 (3.28)	7.29 (1.89)	N/A		2.33		.14	
SIDAS^h^ suicidal ideation score, mean (SD)		6.47 (8.69)	4.69 (10.40)	6.14 (7.76)	N/A		1.80		.17	

^a^GP: general practitioner.

^b^N/A: not applicable.

^c^*P*<.05.

^d^PHQ-9: Patient Health Questionnaire-9.

^e^GAD-7: Generalized Anxiety Disorder-7.

^f^PADIS: Panic Disorder Screener.

^g^SOPHS: Social Phobia Screener.

^h^SIDAS: Suicidal Ideation Attributes Scale.

### Costs of Implementation

Social media advertising costs a total of US $8172; however, half of these advertising costs were directed toward recruiting participants for the control condition. Flyers and posters for clinics and pharmacies cost US $656, along with tablets (hardware and software) to enable screening in these settings cost US $1932.

Staff time for the social media advertising was estimated at 30 minutes per week to oversee advertising and billing for a total of US $734. Staff time for clinics and pharmacies was estimated at 60 minutes per site per week, which included monthly visits to each site to ensure the materials remained visible and the tablets were operational, as well as the travel time and time to maintain relationships and engagement with each site (both during and between site visits). After accounting for travel costs and staff time, these costs were US $7610 for the 5 GP clinics and US $7610 for the 5 pharmacies.

On the basis of the total costs of social media dissemination (US $4820), the cost of disseminating the intervention was US $4.87 per user. The cost of GP clinic dissemination (US $8904) was US $557 per user and that for pharmacy dissemination (US $8904) was US $1272 per user.

### Adherence

Users in the social media pathway completed an average of 2.21 (SD 3.40) modules compared with those in the GP pathway (mean 3.56, SD 4.15) and in the pharmacy pathway (mean 1.14, SD 0.99). The 2-way comparisons between pathways were not significant (social media vs GP: *t*_1005_=1.57, *P=*.12; social media vs pharmacy: *t*_996_=.83, *P=*.41; GP vs pharmacy: *t*_21_=1.51, *P=*.15), although high rates of type II errors were likely because of the small sample sizes.

## Discussion

### Principal Findings

This study compared 3 dissemination pathways for delivering a self-guided internet-based transdiagnostic intervention to reduce the symptoms of depression and anxiety. The findings supported hypotheses 1 and 2, indicating that delivering such interventions directly to end users through web-based marketing in social media leads to considerably greater uptake and reach than dissemination through primary care clinics and community pharmacies. Disseminating internet interventions to users through the same modality as the intervention (ie, social media dissemination) is likely to minimize behavioral gaps between being offered an intervention and engaging with the intervention. The disconnection between the clinic or pharmacy setting and an internet intervention may be an insurmountable gap unless the individual is highly motivated and supported to engage. Despite the low intervention uptake, the results suggest that screening for mental ill health in GP clinics and pharmacies appears to be feasible, with completion rates similar to those in the social media setting. By virtue of the low intervention uptake in GP and pharmacy settings, the per user cost of delivering the intervention was approximately 100 to 250 times greater in these 2 settings than in social media dissemination, supporting hypothesis 3. It appeared that GP users typically completed 1.5 modules more than social media users, although the difference was not significant by virtue of the small sample size. Contrary to hypothesis 4, users from the pharmacy pathway had lower adherence rates.

Exploratory analyses revealed variations in the characteristics of users across the 3 pathways. All pathways showed an underrepresentation of males, greater than would be expected based on the gender-specific prevalence of depression and anxiety [55]. Although web-based social media users tended to be younger (413/991, 41.7% vs 6/23, 26%), age distributions were not significantly different across pathways. GP and pharmacy participants were much more likely to be outside the labor force than the general population; in Australia, 66% of adults were employed, and 5.6% were unemployed [56], with the remainder not in the labor force because of reasons such as retirement or studying. This distribution most closely resembles that of the social media pathway, with working adults underrepresented in the other 2 pathways. Linguistic diversity was underrepresented [57] in all pathways, particularly on social media, which suggests that language or culture may be a barrier to engagement with internet interventions delivered in English.

Finally, there was some indication that anxiety symptoms were less severe among GP clinic participants than in other settings, with significantly lower generalized anxiety scores, although caution is needed because of the small sample size. Although screening for general psychological distress restricted the range of mental ill health in the sample, the findings suggest that participants engaged through GP clinics may have less severe anxiety than those who engaged through social media. Anxiety may be a distinct barrier to engagement with health services, such that recruitment in primary care may miss a sector of the population that experiences high levels of anxiety symptoms. Such differences were not observed for depression symptoms.

This study has several important implications for research and for the dissemination of internet-based interventions in the community. Clearly, for self-guided interventions, dissemination through web-based marketing appears to be considerably more efficient and cost-effective than dissemination through clinics or pharmacies. This may reflect the reluctance of people to engage with an intervention that is seen as distinct from the care they receive from the provider. It is possible that privacy concerns may influence decisions to engage more in public settings than when using the internet in private settings [58]. Furthermore, the web-based modality of the intervention may be more conducive to direct web-based dissemination than via the use of posters. Engaging directly with an intervention through a link may have fewer barriers to participation than requiring typing in a link from a poster, using a QR code from a poster, or using a shared device (noting that these data were collected before the COVID-19 pandemic). In contrast, engaging with a digital service advertised in a GP clinic or pharmacy may be seen by some as having implicit professional endorsement or greater credibility, although such effects were not observed here. Further research to understand the motivations of users across diverse dissemination settings may provide further insights into the barriers to and facilitators of implementation.

Greater success in dissemination in the web-based pathway did not result in significantly poorer adherence or a marked restriction in the diversity of participants. Although it may be slightly challenging to engage older adults or people from linguistically diverse backgrounds through social media marketing, there are methods for attracting specific subgroups through this pathway, such as tailored advertising and targeted platforms or groups. Nevertheless, the success of social media dissemination appears to outweigh these potential challenges, as the number of participants in all subgroups far exceeded those in the other pathways.

### Limitations

There were some limitations to this study. The marketing materials were not consistent across the 3 pathways, with more information provided in the flyers or posters than in the social media advertising. It is possible that other marketing approaches in GP clinics and pharmacies may be more effective. However, we were limited by the constraints of these private, commercial enterprises in the types of marketing we could deliver, and there are few other methods that have been shown to be successful. Although informal testing of marketing materials was conducted before the study, formal testing of how the design of marketing materials influences willingness to participate would be a useful addition to future research. Although the catchment areas for all pathways were matched, the numbers exposed to marketing differed across pathways. Although we were able to estimate and account for these differences, there may have been some minor inaccuracies in these estimates. Furthermore, as noted previously, the way people engage with posters and tablets in a public setting may be different from how they engage with an advertising link in their social media accounts. It is likely that engagement would be considerably higher for interventions that are directly recommended by a clinician rather than those from referral through marketing material within the clinic. Although this study used a passive engagement strategy that was not reliant on staff, it is possible that staff attitudes may have influenced engagement, although these attitudes were not directly assessed. Social media participants were recruited into a randomized controlled trial of the intervention, with the current data only reporting on those participants allocated to the active arm of the trial. Primary care and pharmacy participants were recruited directly to the web-based intervention, with no comparison arm. It is possible that barriers to engagement in trials may have contributed to differences in dissemination outcomes for the social media arm, although these barriers are unlikely to have improved outcomes. Cost estimates excluded the time of staff in clinics and pharmacies. As noted, the low numbers in the GP and pharmacy settings preclude firm conclusions about the differences in user characteristics and adherence. Finally, it is not clear whether these findings would generalize to clinician-guided interventions, non–internet-based interventions, or internet interventions for other health conditions. Involvement of providers in the referral process or characteristics of the health system (eg, costs and attitudes) may also influence engagement with interventions.

### Conclusions

Given the low use of web-based mental health programs but the substantial evidence for their effectiveness, there is an urgent need to improve the uptake of web-based mental health interventions in the general community. This project has identified that a web-based dissemination pathway is the most efficient and cost-effective way of delivering self-guided internet-based mental health programs to people in the community without considerably sacrificing diversity of reach or adherence. Although screening in GP clinics and pharmacies appears to be feasible, in these settings, the behavior gap between completing an assessment and signing up for a digital intervention appears to be considerable.

## Abbreviations

DQ5: Distress Questionnaire 5
GP: general practitioner
